# Integrin Signaling in Mammary Epithelial Cells and Breast Cancer

**DOI:** 10.5402/2012/493283

**Published:** 2012-03-01

**Authors:** Arthur W. Lambert, Sait Ozturk, Sam Thiagalingam

**Affiliations:** Molecular Medicine Program, Biomedical Genetics Section, Department of Medicine, Boston University School of Medicine, 72 East Concord Street, L320, Boston, MA 02118, USA

## Abstract

Cells sense and respond to the extracellular matrix (ECM) by way of integrin receptors, which facilitate cell adhesion and intracellular signaling. Advances in understanding the mammary epithelial cell hierarchy are converging with new developments that reveal how integrins regulate the normal mammary gland. But in breast cancer, integrin signaling contributes to the development and progression of tumors. This paper highlights recent studies which examine the role of integrin signaling in mammary epithelial cells and their malignant counterparts.

## 1. Introduction

The extracellular matrix (ECM)—composed of numerous insoluble proteins secreted locally by epithelial and stromal cells—provides physical support to organize neighboring cells within a tissue and serves as a reservoir of growth factors. In the mammary gland, ECM interactions can control epithelial cell proliferation, survival, migration, and differentiation to regulate processes such as branching morphogenesis, polarization of mammary ducts and the alveolar outgrowth, and involution that occurs with pregnancy [1]. However, the matrix, which constitutes one component of the diverse tumor microenvironment, changes dramatically during the process of breast tumorigenesis and can strongly affect disease progression [2]. Therefore, the ECM can exert a strong influence on both normal and tumor cells.

 In either case, cells sense and respond to the ECM by way of transmembrane integrin receptors, which recognize and bind to various ECM proteins and thereby facilitate cell adhesion and intracellular signaling. Integrins function as a heterodimer, consisting of one *α* subunit and one *β* subunit that associate noncovalently. Mammals have 24 distinct integrin receptors, formed from a total of 18 *α* subunits and 8 *β* subunits [3]. Integrins couple recognition of ECM ligands to the assembly of the actin cytoskeleton and the activation of various intracellular kinases [4]. Here, we review recent studies that have deepened our understanding of the dynamics and coordination of integrin signaling and of the role that these signals play in mammary epithelial cells and their malignant counterparts.

## 2. Integrins in Normal Mammary Epithelial Cells

With a disease as diverse as breast cancer—in its histology, genetic lesions, proliferation, response to treatment, and propensity to metastasize—it is crucial to examine how the cell type that is initially transformed impacts the tumor that is subsequently formed, a concept which emphasizes the cell of origin for a particular cancer [5]. In this regard, new developments in the field of mammary stem cells and advances in understanding the mammary epithelial cell hierarchy have paved inroads for those examining this concept in breast cancer. And, perhaps not surprisingly, integrins have already played a prominent role.

 The epithelium of the mammary gland is composed of luminal cells, which line the ducts and alveoli, and myoepithelial cells which form the basal cell layer that surrounds luminal cells and contacts the basement membrane, a specialized form of ECM rich in collagen IV and laminins [6]. Integrin expression in the mammary epithelial cells is complicated as it is regulated spatially and temporally as the gland develops and through pregnancy, lactation, and involution [7]. However, a few points regarding integrin expression in the mammary gland are useful here. First, mammary epithelial cells are anchorage dependent and require cell-cell interactions or integrin-mediated attachment to the ECM; in the absence of such adhesion, a cell will not proliferate in response to growth factors [8] and will succumb to a specialized form of apoptosis—anoikis—that occurs as a result of detachment from the ECM [9].

**Figure 1:**
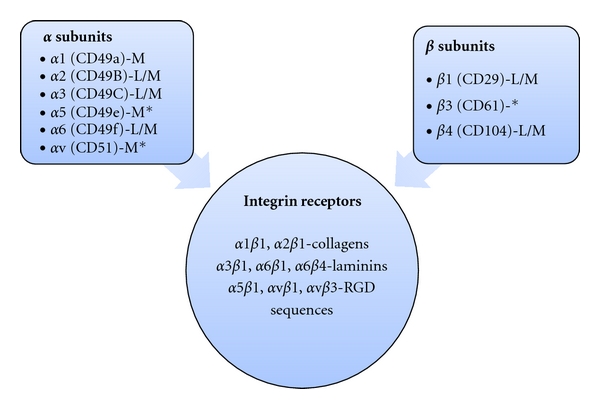
Integrin expression in the normal mammary gland. The *α* and *β* integrin subunits expressed in the mammary gland are listed with the CD alias in parentheses. Their lineage expression pattern is indicated as L (luminal), M (myoepithelial), and L/M (luminal and myoepithelial). *indicates a relatively low-level or restricted expression pattern. The lineage expression of the *β*3 integrin is not clear. The functional integrin receptors and their ligand class are shown below. See [7, 10–12] for more information.

 Second, although integrin expression and activation can vary within the gland, a somewhat limited set of integrins are expressed—as assessed by immunohistochemistry—with certain integrins restricted to either the luminal or myoepithelial cells (Figure 1). In general though, as myoepithelial cells make more extensive contact with the ECM, integrin levels tend to be higher in this lineage [7]. The major *α* subunits expressed include *α*1, *α*2, *α*3, *α*5, *α*v and *α*6; the expression of *α*1, *α*5, and *α*v appears to be restricted to myoepithelial cells [7, 10]. The *β*1 and *β*4 integrin subunits are expressed in both epithelial lineages of the gland, while the *β*3 subunit exhibits a more restricted expression pattern [7, 10, 11]. Thus, epithelial cells of the mammary gland are capable of assembling at least eight functional integrin receptors including two collagen receptors (*α*1*β*1, and *α*2*β*1), three laminin receptors (*α*3*β*1, *α*6*β*1, and *α*6*β*4) and three integrins (*α*5*β*1, *α*v*β*1, and *α*v*β*3) which recognize RGD sequences present in certain ECM molecules such as fibronectin and vitronectin [3]. However, the relative expression level of each complex, their potential interactions with the ECM, and their ability to activate intracellular signaling—in short, their functional status—are not reflected in the basic survey of integrin expression presented above. This information must be derived empirically, as some studies discussed below demonstrate.

 Finally, integrin expression is often polarized, with complexes being localized to distinct membrane surfaces. The best example here is the *α*6*β*4 integrin, which is found predominantly on the basal surface of myoepithelial cells [10]. The *β*4 integrin is unique in that it has a much longer cytoplasmic tail and connects with intermediate filaments in place of the actin cytoskeleton [3]. The localization of the *α*6*β*4 complex—a laminin receptor—to the basal surface allows myoepithelial cells to adhere to the laminin rich basement membrane through specialized adhesive complexes referred to as hemidesmosomes [10]. Nevertheless, simple examination of integrin expression patterns has been of only limited use in understanding their function in the epithelial cells of the mammary gland. And, as our knowledge of the epithelial hierarchy has progressed, a more detailed view of integrin signaling in the mammary gland has come into focus.

 Early experiments involving serial transplantation of mammary epithelial fragments strongly supported the idea that self-renewing stem cells existed in the mammary gland [13]. A significant milestone was reached in 2006 when two reports used cell surface markers to isolate populations enriched for mouse mammary stem cells, in which a single stem cell could reconstitute a functional gland [14, 15]. Two integrins, *β*1 (CD29) and *α*6 (CD49f), were used to purify mammary stem cell populations with a cell surface phenotype of CD29^high^CD24^+^ and CD49f^high^CD24^med^, respectively. Analogous studies have since been performed using human cells and a humanized mammary fat pad transplantation assay and, here too, high expression of *α*6 integrin (along with the absence of EpCAM expression) was used to purify a mammary stem cell enriched population [16]. The scope of these studies was not to address the role of integrins in mammary stem cells, but the results almost certainly indicate that mammary stem cells possess a characteristic set of integrins and suggest that these integrins, and therefore distinct cell-ECM interactions, might have a functional role in regulating the mammary epithelial cell hierarchy.

 This notion has received some support in recent years, mainly through the use of mammary-specific deletion of integrins. But prior to the development of these more refined models, knockout mice had been generated for all of the *α* and *β* subunits present in the mammary gland [3]. Of these mice, the *α*5 [17] and the *α*v [18] are embryonic lethal, restricting their applicability to mammary gland development. Mice with deletion of the *α*1 [19], *α*3 [20], and *α*6 [21] subunits display no apparent mammary phenotype [3, 7], but animals deficient in the *α*2 subunit have impaired mammary gland branching morphogenesis [22]. Knockout of the *β* subunits present in the mammary gland give rise to variable phenotypes ranging from embryonic lethality for *β*1 integrin [23], hemorrhaging and defects in platelet aggregation for *β*3 integrin [24], and severe skin blistering and early death for *β*4 integrin [25] but no overt mammary gland defects. It is likely, though, that the lack of any mammary-specific findings in most of the models described above is masked by the dramatic phenotypes observed during development or in other organ systems, and thus does not discount the relevance of these receptors to the epithelial cells of the mammary gland.

 Early efforts to uncover the function of integrins in the mammary gland utilized function-blocking antibodies directed at *β*1 and *α*6 integrins as well as an antilaminin antibody [26]. These studies found that blocking both laminin and the *β*1 subunit, but not *α*6, impaired ductal morphogenesis, which was in line with a previous study that used a dominant negative *β*1 integrin to demonstrate a role for this subunit in alveolar differentiation [27]. It was surprising then, when rudimentary glands isolated from *α*3, *α*6, or *β*4 knockout mice were able to undergo normal ductal morphogenesis when transplanted into syngeneic recipients [28].

 But with the advent of techniques that allow genetic disruptions to be targeted to specific tissues at defined times during development, it has been possible to more closely examine integrin function in the mammary gland. This is especially true for *β*1 integrin (Table 1), which serves as a subunit for numerous integrin receptors found in the mammary gland. Although conditional mammary deletion of *β*1 integrin—created by crossing mice floxed for *β*1 with transgenic mice that express Cre recombinase from the MMTV promoter—develops normal mammary glands [29], two reports published in 2005 were able to disrupt the *β*1 subunit in a similar fashion, but instead crossed the floxed mice with transgenic mice that express Cre recombinase from either the *β* lactoglobulin (Blg) or whey acidic protein (WAP) promoter [30, 31]. This strategy allowed for conditional deletion of *β*1 integrin in defined epithelial compartments and at certain stages of development; Blg-Cre gives rise to *β*1 deletion in the ductal epithelium in nulliparous mice after puberty, while WAP-Cre generates deletions in luminal alveolar cells during mid-pregnancy. In both cases, deletion of the *β*1 subunit impaired lobuloalveolar development and lactation, which was attributed to inhibition of luminal cell proliferation as a result of increased p21^Cip1  ^[30] and failure to differentiate in response to prolactin [31].

*β*1 integrin has also been deleted from the basal cell population of the mammary epithelia by expressing Cre from the keratin 5 (K5) promoter [32]. Interestingly, this resulted in a depletion of mammary stem cells and produced glands that lacked regenerative potential. Furthermore, dividing basal cells lacking *β*1 integrin aberrantly gave rise to luminal cells. These results expand upon the functions of this integrin subunit in the mammary gland and strongly support the idea that *β*1 integrin expression, and more generally distinct cell-ECM interactions, play a crucial role in regulating the epithelial cell hierarchy of the mammary gland.

 While no conditional *β*4 knockout has been reported, mice with targeted deletion of the C-terminal domain, which allows for prolonged survival and reproduction, appear to develop normally with no mammary gland defects reported [33]. As *β*4 dimerizes with *α*6 integrin, this is in line with studies, discussed above, that found no requirement for *α*6 using function-blocking antibodies or mammary gland transplants from *α*6 knockout mice [26, 28].

 No conditional knockout in the mammary epithelium exists for *β*3 integrin either, although this is likely because the role of this subunit in the gland has only recently been appreciated. A subpopulation of luminal progenitors—with the capacity to differentiate into either ductal or alveolar luminal epithelial cells—has been identified on the basis of *β*3 integrin expression [11]. In this study, the authors found that a subset of luminal cells, which are enriched in the CD29^low^CD24^+^ population [14], express high levels of the *β*3 integrin, form exclusively luminal colonies, and proliferate extensively when grown in Matrigel. The percentage of *β*3 expressing cells decreases with pregnancy, ostensibly as a result of luminal differentiation [11]. This integrin appears to be a reliable and useful marker of luminal progenitors, but similar to the integrins used to purify mammary stem cells, whether these receptors and their downstream signaling pathways confer any distinct functions upon these subpopulations is still an open question.

## 3. Integrin Signaling in Breast Tumorigenesis

### 3.1. Basic Integrin Signaling Mechanisms

Integrin recognition of ECM proteins induces allosteric changes that allow the receptor to transduce this signal across the membrane, a process referred to as outside-in signaling [3]. As integrins possess no enzymatic function, activation of integrin receptors results in the association of multiple protein complexes with the short cytoplasmic tails of the integrins. This allows integrins to transmit mechanical signals to the actin cytoskeleton (and in the case of *β*4 integrin, to intermediate filaments) through proteins such as *α*-actinin, tensin, paxillin, and vinculin as well as biochemical signals by way of tyrosine kinases such as focal adhesion kinase (FAK) or Src [34]. Tyrosine kinase signaling leads to the recruitment of numerous adaptor proteins including p130Cas, Crk, and the IPP complex, which further propagate the signal within the cell [35]. While the effects of integrin signaling are varied and dependent on both the ECM ligand and specific integrin receptor engaged, these receptors function essentially to control cell migration, proliferation, and survival.

 Integrin signaling occurs in cooperation and coordination with growth factor signaling that is initiated from soluble factors in the extracellular environment. This makes sense as epithelial cells are anchorage dependent and require adhesion, mediated by integrins, in order to proliferate in response to growth factors [8]. Crosstalk between integrin and growth factor signaling occurs at many different levels. For one, integrins and growth factor receptors can activate similar downstream signaling pathways such as tyrosine kinases and the MAP kinase pathway, and this combined activation can further amplify the signal [36]. Upon activation, integrins and growth factors receptors cluster together in the membrane and appear to physically interact with each other, which may also help to coordinate signaling [37]. For example, *α*6*β*4 and *α*6*β*1 integrins have been found in association with ErbB2 (HER2), and this interaction may help to potentiate signaling [38]. There is also evidence that integrins can activate growth factor receptors by promoting phosphorylation of the receptor [39] or by stimulating the production and secretion of the growth factor itself [40]. Finally, expression of integrins and growth factor receptors can be coordinated; as an example, *α*6*β*4 can enhance ErbB2 translation [41]. Although many of the mechanisms described here have been elucidated in tumor cells, or cells overexpressing the integrin or growth factor receptor, the coordination of integrin and growth factor signaling is well established. And while the dynamics of these signaling networks that operate in normal mammary epithelial cells are less clear, the integrin and growth factor pathways are inextricably linked as is evident from morphological and functional studies of the mammary gland [10, 42].

 Integrin receptors are unique in that they can also transmit signals emanating within the cell to the extracellular environment in what is known as inside-out signaling [3]. In this mode of signal transduction, interactions from the cytoplasmic tail are conveyed to the extracellular domain through conformational changes. This type of signaling is especially important when integrin activation must be strictly regulated, as in the case of platelets [3]. In epithelial cells, however, this bidirectional communication allows integrins to control remodeling of the ECM [43]. Furthermore, since the matrix proteins bind numerous growth factors and regulate their availability, reorganization of the ECM by integrins can liberate occult soluble signals [44].

### 3.2. Deregulation of Integrins in Breast Cancer

Integrins are not considered to be bona fide oncogenes or tumor suppressors but their expression levels are affected by transformation [45] and breast cancer cells exhibit altered levels of integrin expression [46]. No characteristic integrin expression pattern can be ascribed to all breast tumors, and it is likely that different subtypes of breast cancer [47] may generate tumors with distinct integrins. Some support for this idea comes from recent studies [16, 48, 49] that have identified luminal progenitors—defined in part by their expression level of *α*6 integrin [16]—as the transformed cell of origin which gives rise to basal-like tumors. Nevertheless, certain integrins are commonly deregulated in breast cancer. On one hand, the *α*6 [50], *β*4 [51], and *α*v [52] integrins are generally overexpressed in aggressive breast cancer cells. On the other hand, expression of *α*2*β*1 can be suppressed during the process of transformation [53]. Ultimately though, it is the function of integrins in tumors cells that is most relevant. Below, we highlight some recent advances pertaining to integrin function in breast cancer cells.

### 3.3. Integrin Signaling in Tumor Initiation

Integrins have begun to emerge as key players in breast tumor initiation. Based in part on the cooperatively of integrin and growth factor signaling described above, a mammary specific deletion of *β*1 integrin was generated in a transgenic mouse model of breast cancer in which the polyomavirus middle T antigen (PyVmT) oncogene, an activator of many of the same signaling pathways downstream of growth factor receptors [54], is expressed from the MMTV promoter [29]. Here, loss of *β*1 integrin severely impaired tumorigenesis indicating a crucial role for this integrin in cell transformation and the initiation of mammary tumors that arise in this model. In line with this, deletion of *β*1 impaired cell proliferation and FAK phosphorylation, which was shown to mark hyperplastic regions of the gland [29] and is known to regulate entry into the cell cycle [55].

 Similar findings have been described for *β*4 integrin in an ErbB2 overexpressing mouse model of breast cancer [56]. In this case, deletion of the *β*4 cytoplasmic domain, which suppresses intracellular signaling, resulted in an increased latency and reduced aggressiveness of ErbB2-induced tumors. A protein complex containing both ErbB2 and *α*6*β*4 integrin could be detected, and this amplified downstream activation of the transcription factors c-Jun and STAT3 to facilitate cell proliferation and suppression of apoptosis. Further, *β*4 integrin signaling enhanced resistance to the ErbB2 inhibitor Iressa, presumably as a consequence of enhanced oncogenic signaling [56].

 The results of these studies support two key points: (1) integrin and growth factor signaling collaborate *in vivo* during tumorigenesis and (2) integrins actively participate in the process of tumor initiation. It is worth noting, that several factors downstream of integrins have also been deleted in the context of the MMTV-PyVmT model of mammary tumorigenesis. Although ablation of FAK decreased proliferation of tumor cells, it only moderately increased the latency of tumor development, and FAK was not required for the generation of mammary tumors [57]. However, it does seem to have a role in tumor progression as cancer cells lacking FAK were not able to metastasize [57]. Deletion of Src in the same tumor model has similar effects including delayed tumor onset, proliferation and cell cycle defects, and impaired tumor progression [58]. The discrepancy between these studies and the *β*1 and *β*4 deletions suggests that integrins may use distinct signaling pathways at different times during tumorigenesis to regulate both tumor initiation and tumor progression.

### 3.4. Integrin Signaling in Cancer Stem Cells

An exciting area of breast cancer research is the study of cancer stem cells. The cancer stem cell hypothesis predicts that a certain subpopulation of tumor cells—first identified for breast cancer in 2003 [59]—drives tumor growth, progression and recurrence [60]. This theory has strong implications for the treatment of cancer, and so there has been a concerted effort to understand the signals which maintain this population in hopes of developing strategies to therapeutically target this tumorigenic population [61]. Recent work has suggested that integrin signaling may have a functional role in the cancer stem cells [12].

 In 2008, a study of various mouse mammary tumor models found that MMTV-Wnt1 mice exhibited aberrant regulation of mammary epithelial cell populations—as defined by CD29 (*β*1 integrin) and CD24 [14]—prior to tumorigenesis with expansion of the CD29^high^CD24^+^ population of mammary stem cells [62]. Importantly, *β*3 integrin expression marked a population of cancer stem cells that was highly tumorigenic. These results imply that Wnt1-induced tumorigenesis arises from an altered pool of luminal progenitors [62], but similar to normal luminal progenitors [11], it remains to be seen whether signaling from *β*3 integrin is relevant in this context.

 Interestingly, FAK has also been implicated in the control of mammary and cancer stem cells. In a separate study where FAK was deleted in MMTV-PyVmT mice, the authors found a reduced pool of mammary stem cells that exhibited defects in self-renewal that were associated with impaired tumorigenesis [63]. This finding is especially interesting in the light of the fact that *β*1 integrin is also involved in mammary stem cell regulation [32] and seems to indicate a key role for integrin signaling in stem cells, which may be coopted during tumorigenesis [64]. It would be interesting then to test the role of *β*1 integrin and FAK in the MMTV-Wnt1 model, where a defined cancer stem cell population has been identified [62].

## 4. Integrins and Breast Cancer Progression

### 4.1. Integrins Signaling in Migration and Invasion

In cancer, integrins are perhaps best known for their role in cell migration and tissue invasion [65]. Migration and invasion are a prerequisite for the complex process of metastasis which is thought to occur through a series of steps involving local tissue invasion, intravasation, survival in the circulation, extravasation, and colonization [66]. The ECM of the mammary gland serves as an inherent barrier to tumorigenesis and metastasis, so tumor cells must find ways to circumvent this suppressive force [67]. As integrins interact directly with the ECM and can control remodeling of the matrix, tumor cells can benefit from altered integrin expression and signaling in the context of metastasis. To metastasize, tumor cells must first be able to migrate. Integrins surely play a role in the process of cell migration and likely function at two different levels. First, integrins must coordinate and stabilize adhesion to the ECM in such a way that allows for cell movement, forming strong adhesive complexes at the leading edge of the cell while releasing previous points of contact at the trailing end [68]. At another level, integrins couple actin reorganization to cellular signals that regulate motility through activation of PI3-kinase and small GTPases such as Cdc42 and Rac [69]. Other integrin signaling pathways are important as well; for instance, FAK is required for epidermal growth factor-induced cell motility [70].

 In addition to their central role in cell motility, integrins have recently been shown to actively participate in the transition of a nonmotile tumor cell to an invasive, malignant cell. Through a process referred to as epithelial-mesenchymal transition (EMT), tumor cells can gain access to signaling programs, normally reserved for development, that allow epithelial cells to migrate and invade the surrounding tissue [71]. EMT can be induced in susceptible breast cancer by the cytokine TGF-*β* [72], but studies have shown that *β*3 integrin signaling and Src activation are required for this occurrence in mammary epithelial cells [73]. Other integrins, with a less established role in breast cancer, such as *α*v*β*6 integrin, have been shown to regulate the release of active TGF-*β* from the ECM [44], but the relevance of this to breast cancer might be limited.

 The induction of EMT is associated with the expression of matrix metalloproteinases (MMPs) that can degrade and remodel the ECM [74]. The *α*v*β*3 integrin, which is overexpressed in certain breast cancers [52], can interact with and enhance the activation MMP-2 in melanoma cells to promote invasion [75], and a similar mechanism could benefit breast cancer cells which express the same combination of proteins. Alternatively, *α*v*β*3 may enhance the expression of MMP-2 [76]. The association of *α*v*β*3 with the urokinase plasminogen activator receptor (uPAR) provides yet another example of how integrins can modulate degradation of the ECM to facilitate breast cancer invasion [77].

### 4.2. Integrins and Metastasis

An association between the expression of certain integrins and the metastatic spread of breast cancer has been suspected for some time. Of note here is the association between high *α*v*β*3 expression and bone metastasis [78], the reduced expression of *α*2*β*1 observed in poorly differentiated adenocarcinomas [79] and the correlation of high *α*6 expression with reduced survival [50]. However, recently, new mechanisms by which integrins regulate metastasis have been elucidated.

 Early efforts to explain the connection between *α*v*β*3 expression and metastasis found that activation of this integrin could help breast cancer cells adhere to platelets, an interaction which may help disseminated cancer cells arrest in the circulation prior to extravasation [80]. However, this integrin receptor may exert a more direct effect on tumor cells by activating intracellular signals, in the absence of cell adhesion, that promote tumor progression [81]. In line with previous studies, the authors observed that expression of this integrin was higher in lymph node metastases and found evidence to suggest that this was a result of enhanced tumor cell survival [81]. Most surprising though, they found that unligated *α*v*β*3 promoted anchorage-independent growth, and presumably tumor progression, through a mechanism that involves recruitment of Src and phosphorylation of CAS. This finding is especially interesting as it suggests that tumor cells utilize integrin signaling independent of cell adhesion and it has clinical relevance as many *α*v*β*3 inhibitors target the ligand binding, which is apparently dispensable here [81]. However, neither of these studies explain why *α*v*β*3 expression is preferentially retained in breast cancer cells that metastasize to the bone.

 New information also highlights the extensive crosstalk between integrins and other oncogenic proteins that are active in tumor cells. A recent report described a new mechanism by which mutant p53 can contribute to cancer cell invasion and metastasis [82]. This study found that mutant p53 promotes increased endocytic trafficking of EGFR and *α*5*β*1 integrin to the plasma membrane through Rab-coupling protein (RCP), which leads to enhanced integrin and growth factor signaling that is required for cell migration and invasion. These results support the recent finding that RCP is oncogenic in mammary epithelial cells [83] and suggest that integrin signaling might be required for RCP to function in this manner.

 Integrins can also collaborate with inhibitor of apoptosis (IAP) proteins to promote breast cancer metastasis [84]. While IAPs are typically thought of as antiapoptotic proteins it appears that their functions are more varied than originally thought [85]. Building upon this, is the recent finding that an XIAP-survivin complex can also regulate tumor cell invasion and metastasis, independent of its antiapoptotic effects [84]. IAPs are able to do this by activating NF-*κ*B, which induces the production of fibronectin. Secreted fibronectin can then act in an autocrine or paracrine to stimulate *β*1 integrin signaling through FAK and Src [84].

 It is important to note that not all integrins promote tumor progression and metastasis. For instance, a recent report has clearly established that the *α*2*β*1 integrin is a metastasis suppressor in breast cancer [86]. Deletion of *α*2*β*1 integrin in the MMTV-ErbB2 mouse significantly increased the incidence of metastasis without affecting growth of the primary tumor. This seems to occur at the level of cancer cell intravasation as tumors devoid of *α*2*β*1 produced increased numbers of circulating tumor cells while tail vein injection—a test of tumor cell extravasation and colonization—of the same cells did not affect metastasis [86]. Using clinical databases of gene expression, reduced *α*2*β*1 expression was strongly correlated to metastasis and decreased survival [86], but the molecular basis underlying this effect remains to be established.

## 5. Concluding Remarks

**Figure 2:**
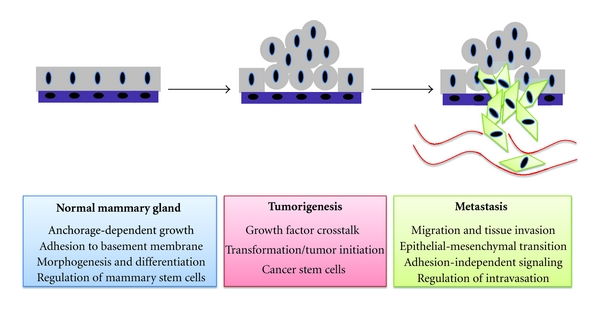
Roles of integrin signaling in mammary epithelial cells and breast cancer progression.

Integrins have a long and storied history in the breast cancer literature and clearly their central role as sensors and transducers of ECM signals is without question. But it has taken some time—with the diverse array of integrins and ECM ligands—to fully appreciate their function. This paper has touched only upon the cell-autonomous functions of integrins in epithelial cells of the mammary gland and during breast cancer progression (Figure 2), but their expression in other cell types such as endothelial cells, fibroblasts and lymphocytes also contributes to tumorigenesis [87]. Given their role in breast cancer and the availability of integrin antagonists, preclinical studies and clinical trials are currently underway to test the efficacy and tolerability of these agents [88]. However, important questions regarding integrin signaling still remain. How the ECM changes that occur with cancer development and disease progression affect integrin signaling in tumor cells is an important area of focus. This would seem to be especially true in the context of metastatic colonization [89], where tumor cells encounter an entirely new ECM and are still able to thrive; surely integrins must have a crucial function here. The relevance of integrin signaling in normal mammary stem cells, luminal progenitors, and other mammary epithelial cell populations is still largely unknown, but it is likely that integrins are involved in regulation of this hierarchy and therefore, perturbed integrin signaling may have distinct effects in different subtypes of breast cancer. For it is only by defining the precise role of integrin signaling in the normal mammary gland that we will be able to appreciate the true extent of its contribution to breast cancer.

## Figures and Tables

**Table 1:** Targeted deletions of **β*1* integrin in the mammary gland.

Cre promoter	Phenotype	Reference
MMTV	No effect on early gland development	[29]
whey acidic protein (WAP)	Disorganized alevoli; inhibited luminal cell proliferation	[30]
**β** lactoglobulin (Blg)	Impaired alveolar morphogenesis and differentiation	[31]
keratin 5	Depletion of mammary stem cells; impaired regenerative potential	[32]
